# Role of Kupffer Cells in Driving Hepatic Inflammation and Fibrosis in HIV Infection

**DOI:** 10.3389/fimmu.2020.01086

**Published:** 2020-06-16

**Authors:** Lumin Zhang, Meena B. Bansal

**Affiliations:** Divison of Liver Diseases, Icahn School of Medicine at Mount Sinai, New York, NY, United States

**Keywords:** liver fibrosis, HIV - human immunodeficiency virus, hepatic stellate cell (HSCs), Kupffer cells, microbial translocation

## Abstract

While the interactions between HIV and various liver cell populations have been explored, the relevance of these interactions when patients are well-controlled on ART is less clear. Therefore, we focus this perspective on HIV-related alterations that may drive hepatic inflammation and fibrosis in aviremic patients, with a focus on Kupffer cells and Hepatic Stellate Cells. Persistent CD4+ T cell depletion in the gut resulting in increased gut permeability has been postulated to play a role in systemic immune activation in HIV patients. The liver, with its unique location, remains the gatekeeper between the gut and the systemic circulation. The resident liver macrophage, Kupffer cell, is responsible for clearing and responding to these products. We propose that changes in Kupffer cell biology, in the context of HIV infection, creates a mileu that drives hepatic inflammation and fibrosis in response to microbial translocation. Targeting these pathways may be helpful in improving liver-related outcomes in HIV patients.

## Introduction

End-stage liver disease is a major cause of non-AIDS related mortality in HIV+ patients even with effective anti-retroviral therapy, accounting for almost 15% of deaths (1–7). As a result of shared routes of transmission, HCV and HBV are the most common liver diseases in HIV-infected patients, although other chronic liver diseases are emerging (8, 9). Most data, therefore, regarding fibrosis progression rates is derived from those with coinfection. These patients have a higher relative risk (RR) of cirrhosis, increased development of decompensated cirrhosis and accelerated fibrosis progression rates compared with those who are only infected with HCV or HBV (10, 11). Furthermore, rapid fibrosis correlates with reduced CD4+ T cell counts and detectable plasma HIV levels. HIV patients are also more susceptible to other liver diseases, which synergize to accelerative liver fibrosis. Alcohol consumption is associated with increased relative risk of fibrosis progression in HIV mono-infected patients (12) while NASH is emerging as a major cause of liver disease, with half of mono-infected patients with unexplained liver enzyme elevations having NASH (13). While many may have an unrecognized chronic liver injury, a higher frequency of liver fibrosis was demonstrated in HIV-1–monoinfected patients (range 11–40.9%) compared with uninfected patients even without coinfection of hepatitis viruses and alcohol abuse, suggesting a correlation between HIV-1 infection and advanced liver fibrosis (14–19). Therefore, persistent HIV-1 infection and viral associated liver immune dysfunction may independently contribute to the progression of liver diseases (20). Lastly, in those on ART, drug-induced liver injury and increased rates of NASH due to both medications and metabolic derangements common in HIV are being observed. While hepatic stellate cells are the downstream effector of liver fibrosis, this perspective focuses on the role Kupffer cells play in promoting a mileu conducive to fibrosis progression in patients with HIV infection, particularly in aviremic patients.

## The Liver as the Gatekeeper

Shortly after HIV infection, a severe CD4+ T cell depletion in the gut-associated lymphoid tissues leads to a disruption of the intestinal barrier, consequently promoting translocation of microbial products into the portal circulation. The liver, which derives the majority of its blood flow from the portal circulation, is uniquely positioned to protect the systemic circulation from gut-derived products. In particular, the resident hepatic macrophage, the Kupffer cell, located within the hepatic sinusoid is charged with clearing translocated bacterial products in an immunotolerant manner. However, when products provoke a pro-inflammatory response by Kuppffer cells, a cascade of intrahepatic inflammatory respones is initiated with numerous secreted cytokines, such as IL-1β, TNF-α, and IL-6, serving as major drivers in the progression of liver injury and fibrosis.

## Kupffer Cells at the Nexus of Liver Inflammatory Responses

Kupffer cells (KCs) are the largest population of resident tissue macrophages in the liver. They reside within the hepatic sinusoid in close proximity to hepatic stellate cells, liver sinusoidal endothelia cells, and intrahepatic lymphocytes. Both the low flow state of the portal circulation and the uniquely fenestrated endothelium create a conducive environment for interaction of KCs with neighboring cells and circulating cells of the immune system. Physiologically, KCs are the first line of defense to eliminate macromolecules, immune complexes, senescent cells, virally-infected cells, and translocated microbial products from the gut to avoid liver injury and systemic immune responses (21). Given the dynamic nature of cell surface receptor expression on macrophage populations and some controversy regarding their origins, CD163 or CD68, CD14 and CD16 are often used to identify human KCs. However, murine KCs display phenotypic patterns characterized by F4/80^+^, MHCII, and CD11b^Int^ expression. A detailed discussion of markers for various macrophage subpopulations within the liver is beyond the scope of this perspective and discussed elsewhere (22). The focus of this perspective is on the role of CD68+human KCs in promoting liver inflammation and fibrosis in patients with HIV.

The importance of KCs in liver injury and inflammation have been established with depletion studies wherein GdCl_3_ was associated with AST reduction and inflammation in an alcohol model of liver injury (23). Crosstalk between KCs and HSCs is also evidenced by KC depletion as mRNA levels of TGF-β, α-SMA and collagen I are significantly decreased (24). Although GdCl3 is not specific to KCs, and thus interpretation is complex, GdCl3 treatment dramatically decreased cytokines predominantly produced by KCs, TNF-α, IL-6, and IL-1β, in response to LPS stimulation in murine livers (25, 26). Similarly, liposome/clodronate can suppress pro-inflammatory responses through the depletion of KCs (27).

In homeostasis, KCs are central to intrahepatic immune tolerance through an antigen-mediated induction of functional arrest of CD4 cells and regulatory T cells. However, in an inflamed microenvironment this delicate equilibrium is disrupted resulting in immune dysfunction and tolerance break (28). Indeed, knockout of TREM-1 (Triggering receptor expressed on myeloid cells), which is highly expressed on KCs in liver fibrosis, reduced liver fibrosis through the inhibition of TNF-α and IL-6 responses in a number of chronic injury models (29). Similarly, knock down of Jun N-terminal kinase $\frac{1}{2}$ (JNK-1/2) from KCs reversed liver fibrosis in a choline-deficient L-aminoacid-defined (CDAA) model, with a decline in inflammatory responses, including TNF-α, IL6, IL-1β, and TGF-β (30).

While KCs display M1-like features in acute liver injury, with protracted chronic inflammation, due to exhaustion of M1-like macrophages and immune cells, M2-like macrophages emerge and secrete protective cytokines upon chronic cytotoxic stimulation such as IL-4, IL-10, and TGF-β (31, 32). IL-10, an anti-inflammatory cytokine, down-regulates macrophage effector functions and differentiation of neighboring cells to maintain immune microenvironment homeostasis. For example, administration of IL-10 decreased TNF-α produced from LPS-treated KCs (33). While very complex, the manipulation of KC mediated immune responses or approaches to limit their stimulation may be exploited therapeutically.

## Microbial Translocation and Kupffer Cells

The impact of translocated microbial products on KCs is well-established. pretreatment with 2.5% dextran sulphate sodium (DSS) causes increased intestinal permeability and promotes translocation of microbial products into the portal blood in mice. The resulting amplified TLR4 mediated inflammatory responses in KCs resulted in significant livery injury (34). Using a liver slice model, LPS stimulation increased IL-1β and TNF-α production compared to the control (35). Consistently, in mouse models, LPS administration rapidly induces the release of inflammatory cytokines in the liver with a higher IL-6 production obtained from LPS stimulated KCs than splenic and alveolar macrophages (36).

## The Role of TLR4 Signaling in Inflammatory Responses of Kupffer Cells

TLR4, as one member of Toll-like receptors, belongs to the pattern recognition receptor (PRR) family. After stimulation by TLR4 ligands, for example lipopolysaccharides, TLR4 is activated through conformational changes and interaction with TIR-domain-containing adapter proteins via hydrophilic interactions. Intracellular TLR4 signaling is mediated by two classical pathways: the TIRAP–MyD88-NF-κB pathway and the TRIF–TRAM-interferon regulatory factor-3 (IRF3)-NF-κB pathway. TLR4 signaling participates in the initiation of pro-inflammatory response, especially TRIF mediated TNF-α and synthesis of chemokines and have been reveiwed in detail elswhere (37).

In addition to TNF-α, TLR4 signaling also contributes to the transmission of two priming signals for the IL-1β pathway through the NLRP3 inflammasome. IL-1β is a crucial proinflammatory cytokine in response to microbial infection. IL-1β from LPS-treated KCs can produce a deleterious effect on hepatocytes and promote the secretion of VLDL apo B and lipid (38). IL-1β was also found to inhibit IFN-α induced STAT1 activation in hepatocytes, attenuating the innate immune response to viral infection in hepatocytes (39). In general, NLRP3 mediated-cleavage of caspase 1 is the critical step to promote the maturation of IL-1β. The formation of the NLRP3 inflammasome is initiated by ATP or microbial stimulation (40). Blockage of NLRP3 activation in KCs decreased IL-1β response to Ischemia/Reperfusion induced liver injury and improved survival (41, 42). Administration of MCC950, a small molecule selective inhibitor of NLRP3, suppressed LPS primed IL-1β response in NPC cells, subsequently, decreasing liver injury (43). Given the important role of TLR4 signaling in KCs, the modulation of this pathway in the context of HIV infection and persistent microbial translocation is critical.

## Modulation of Inflammatory Responses by HIV-1 Infection in KCs

In addition to CD4, both CCR5 and CXCR4, HIV-1 co-receptors, are detected on human KCs isolated from non-HIV-1 individuals, suggesting that KCs are permissive for HIV-1 infection. HIV-1 infection of KCs in viremic patients has been shown by *in situ* hybridization for HIV-1 RNA and PCR for proviral DNA on FACS-purifed KCs from livers of patients with Acquired Immunodeficiency Sydnrome (AIDs) (44–46). Moreover, retrieval of HIV-1 from KCs derived from patients either not on ART (47) or on ART for short durations has been shown and supported by studies in SIV_DH12R_-infected macaques (48, 49). Recently it has been shown that KCs derived from patients on long term ART, while containing evidence of HIV-1 transcripts, do not secrete replication competent virus (50). While macrophages are known to be able to transmit infectious virus to susceptible CD4+ cells via cell-cell contact (51, 52), the ability of KCs in patients on long-term ART to do so has not yet been explored though warrants investigation.

We have shown that Kupffer cells are highly permissive for HIV-1 infection *in vitro* with robust and sustained viral replication (53). HIV-1_BaL_, a laboratory adapted CCR5-tropic HIV, infection rendered KCs more sensitive to LPS treatment through an increase in CD14 and TLR4 expression on the cell surface, resulting in increased secretion of TNF-α and IL-6, which was blocked by a small molecule TLR4 inhibitor. Interestingly, despite AZT and ritonavir abrogated viral replication, KCs maintained their sensitivity to the pro-inflammatory response to LPS. These findings suggest that even in patients on ART, KC biology may be impacted and promote a mileu supporting hepatic inflammation and fibrosis in response to microbial translocation. While no change in IFNα or IFNβ expression in HIV-1 infected KCs was observed, IL-1β mRNA and both intracellular and secreted IL-1β was increased by HIV-1_BaL_ infection. Similar to the IL-6 and TNF-α response, this HIV-related sensitization was found to be TLR4-dependent and further determined to be via the NLRP3-caspase 1 pathway. Immunostaining on liver tissue derived from aviremic HIV+ patients demonstrated an increased expression of IL-1β compared to normal liver with a high degree of colocalization in CD68+ macrophages (54). These studies show that TLR4 mediated NLRP3 activation is critical for the inflammatory responses to microbial products in KCs. Importantly, liver injury and resulting damage-associated molecular patterns (DAMPS) also activate TLR4 signals in KCs and thus may play a role in other forms of liver injury in HIV patients such as drug-induced liver injury. Interestingly, it has also been shown that CCR5 and TLR may co-cluster on monocyte-derived macrophages (MDMs) as secretion of CCL2 and CXCL8 in response to either R5 gp120, recombinant envelope protein from CCR5-tropic HIV-1, or LPS can be blocked by either a CCR5 inhibitor or TLR4 blocking. These results suggest another mechanism for synergistic effects of HIV and LPS on macrophage biology and should be specifically examined in human KCs (55).

## Inflammatory Responses to HIV-1 Infection in Other Liver Immune Cells

While beyond the scope of this perspective, HIV-1 infection impacts a number of other cells critical to the inflammatory response in the liver. In line with circulating CD4+ T cells, HIV infection leads to a depletion of CD4+ T cell in the liver with relative reversal of CD4/CD8 ratio typically seen. Viral infection also makes IL2+ CD4+ T cells dysfunctional and attenuates hepatic immune response to microbial infection (56–58). CD4+ T cells from HIV mono-infected patients exhibit a low regulatory effect on Natural killer (NK). Co-cultured with NK cells, CD4+ T cells from HIV-1+ individuals greatly reduced anti-fibrotic effect of NK cells on HSCs (59). Therefore, reduction in CD4+ T cells influences progression of liver fibrosis in HIV+ patients (10, 60) while increased relative CD8+T cells correlates with a higher fibrosis scores in HIV-1 infected patients (61).

NK cells, which account for up to 30–50% human liver lymphocytes, play an important role in clearing virally infected cells trough NK cell antibody dependent cell cytotoxicity (ADCC). The activation and NK cellular numbers are spontaneously increased early in response to HIV-1 infection but with chronic infection exhaustion results in NK dysfunction with persistent viremia (62).

While the role of DCs in HIV-1 infection and progression and ability to transmit infectious virus to CD4 cells by cell-cell contact has been shown, HIV interaction with DCs in the liver is less studied. TLR7 is constitutively expressed by human pDCs. The delivery of HIV-1 viral nucleic acids in early endosome of pDCs can be blocked by TLR7 inhibitor (63), suggesting that TLR7 is involved in the antigen presentation by pDCs. In addition, mDC express TLR4 and the frequency of mDCs, especially CXCL16-producing mDCs has been shown to be associated with the level of microbial products in the liver of HIV+ patients (64).

## Interactions Between HIV and Hepatocytes and Implications for Hepatic Inflammation and Fibrosis

*In vitro* studies have shown that the envelope protein, HIV gp 120, which binds either CXCR4 (X4) or CCR5 (R5) on its target cell can promote hepatocyte apoptosis (65) and along with the HCV glycoprotein E2 promote the secretion of the pro-inflammatory cytokine IL-8 (66, 67). It has been known that Kupffer cells play a primary role in the clearance of apoptotic hepatocytes/cellular debris within the liver and thus play a key role in sterile inflammation and repair (68). More recently, Ganesan et. al demonstrated that ethanol exposure promotes HIV accumulation within hepatocytes, ultimately leading to increased oxidative stress and apoptosis. These apoptotic hepatocytes then stimulate inflammasome activation in KCs and pro-fibrogenic genes in hepatic stellate cells (69). Moreover, hepatic stellate cells can also engulf apoptotic hepatocytes resulting in NAPDH oxidation, stellate cell activation, and fibrogenesis (70). Therefore, effects on HIV on hepatocytes can promote both KC and HSC activation, synergistically driving hepatic inflammation and fibrosis.

## Direct Interactions Between HIV and Human Stellate Cells

HSCs express both HIV CCR5 and CXCR4 co-receptors. We have shown that HIV and its envelope protein gp120 promote HSC activation, collagen I production, and CCL2 secretion through interactions with CXCR4 (71) and others have shown that the envelope protein on HIV that preferentially uses CCR5 for cellular entry (R5 gp120) promotes HSC chemotaxis and CCL2 secretion (72). While HIV can infect HSCs *in vitro*, infection *in vivo* has not been established. Similar to what has recently been shown for KCs, *in vitro* infected HSCs do not secrete replication competent virus though, like DCs, may be able to transmit virus by cell-cell contact (71, 73, 74). Similar to what has been shown on MDMs, CCR5 and TLR4 seem to co-cluster on HSCs and result in increased CCL2 and CXCL8 in response to gp120 and LPS, with effects of ligands blocked by inhibiting either receptor alone (55). As CCL2 is an important chemokine for attracting circulating monocytes into the liver, this may be important for propagating hepatic inflammation. R5 gp120 also promotes IL-6 secretion from HSCs through Jun-NF-kB activation (75). These studies suggest that HIV promotes inflammation and fibrosis by interacting with CXCR4 and CCR5 via gp120 and synergizes with TLR4 signals. While the latter is important in viremic patients, relevance for those on ART are not clear. For those on ART, the impact of HIV on microbial translocation and KC biology may be more important.

## Interplay Between Kupffer Cells and Hepatic Stellate Cells

**Figure 1:**
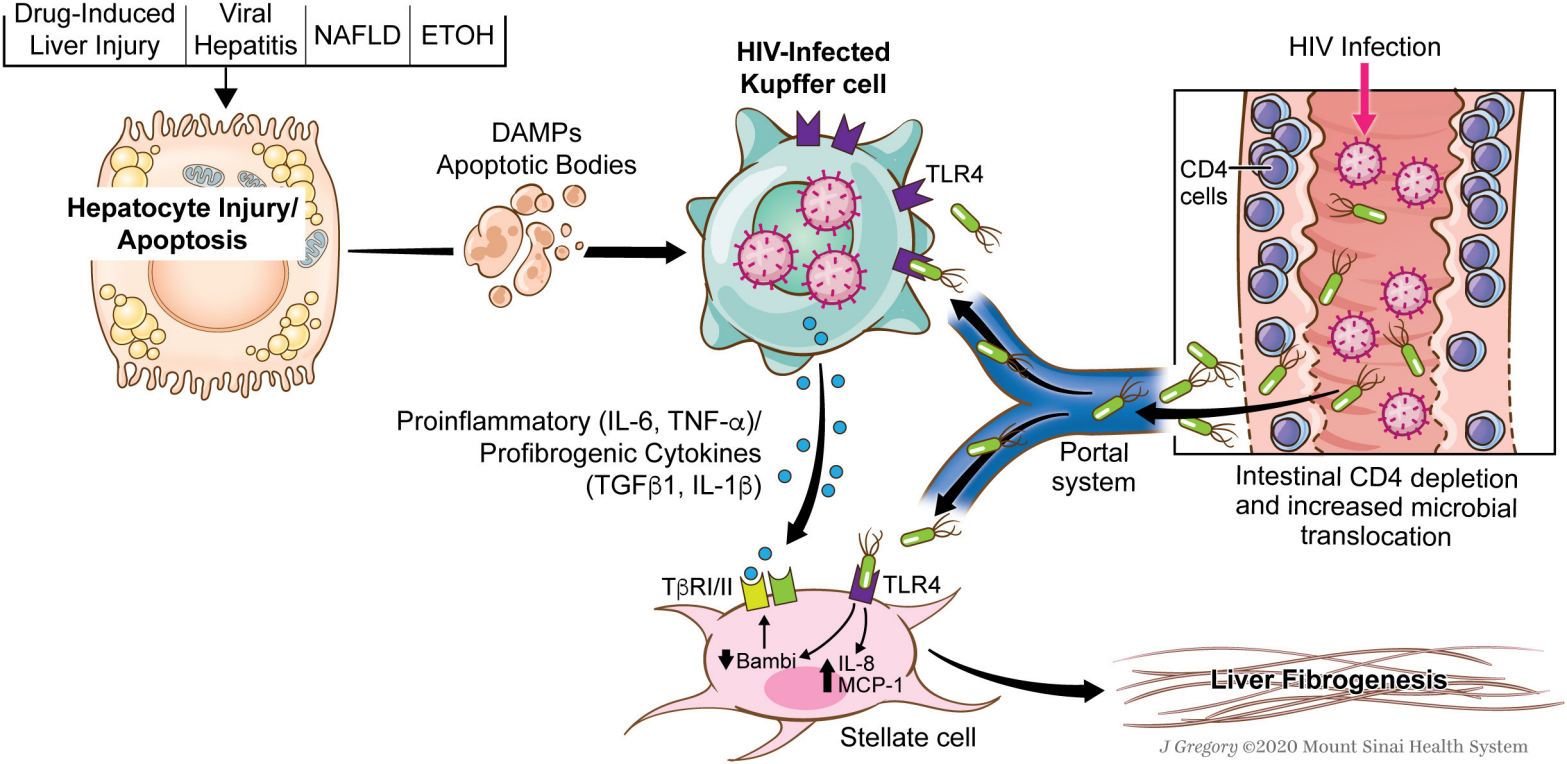
Role of HIV and associated microbial translocation in driving hepatic inflammation and fibrosis through kupffer cell and stellate cell interactions. HIV infection causes early CD4 gut depletion which promotes increased microbial translocation and the delivery of pathogen-associated molecular patterns to the liver (PAMPs). At the same time, patients with HIV have multiple secondary chronic liver injuries (NAFLD, DILI, HCV, HBV, Alcohol) which promote direct injury to hepatocytes and give rise to damage-associated molecular patterns (DAMPs) or endogenous TLR ligands. HIV infected KCs are sensitized to effects of both the translocated microbial products as well as DAMPs. These signals converge on the HIV-infected KCs resulting in the secretion of a number of pro-inflammatory (IL-6, TNF-α) and pro-fibrogenic cytokines (IL-1β and TGFβ1). Additionally, TLR ligands have pro-inflammatory effects on HSCs and sensitize them to KC-derived TGFβ1. Used with permission from @Mount Sinai Health System.

While the ultimate effector cell in liver fibrosis is the hepatic stellate cell, the signals generated by KCs are critically important in promoting the activation of HSCs and then perpetuating the activated state. TLR4 activation on HSCs results in downregulation of the TGFβ1 pseudoreceptor, BAMBI, which sensitizes HSCs to the pro-fibrogenic effects of TGFβ1 (76), much of which is derived by KCs. Therefore, in the context of HIV-1 and associated microbial translocation, the effects on both KCs and HSCs are compounded and drive hepatic inflammation and fibrosis (Figure 1). Overall association between microbial translocation and liver fibrosis progression has been shown in a variety of liver diseases and thus HIV simply compounds this effect.

## Conclusion

As patients with HIV live longer, liver disease will continue to emerge as a leading cause of morbidity and mortality. Understanding how HIV may set the stage for hepatic injury, inflammation and fibrosis may lead to novel therapeutic strategies. While treatment of underlying diseases, ranging from viral hepatitis to NASH or alcohol, remains the most imporant strategy, the alterations unique to this population need to be kept in mind. With this perspective, HIV related alterations in KC biology and microbial translocation may be at the nexus of creating a milue conducive to hepatic fibrosis. Targeting either the KC response to TLR4 ligands, PAMPs or DAMPS, decreasing the burden of microbial products from reaching the portal circulation, or blocking downstream pro-inflammatory or pro-fibrogenic effects on stellate cells are important to consider. Much will be learned from current treatments undergoing investigation for non-HIV related liver fibrosis that may be additionally leveraged for this special population.

## Author Contributions

LZ and MB wrote and reviewed this manuscript.

## Conflict of Interest

The authors declare that the research was conducted in the absence of any commercial or financial relationships that could be construed as a potential conflict of interest.
